# Pain Perception in Phacoemulsification with Topical Anesthesia and Evaluation of Factors Related with Pain

**DOI:** 10.4274/tjo.13914

**Published:** 2016-08-15

**Authors:** Zeynep Dadacı, Mehmet Borazan, Nurşen Öncel Acır

**Affiliations:** 1 Mevlana University Faculty of Medicine, Department of Ophthalmology, Konya, Turkey

**Keywords:** pain, phacoemulsification, topical anesthesia, verbal pain scale, visual analog scale

## Abstract

**Objectives:**

Evaluation of pain during and after phacoemulsification with topical anesthesia in patients with senile cataract and investigation of factors related with pain.

**Materials and Methods:**

Ninety-two adult patients scheduled for routine clear corneal phacoemulsification with topical anesthesia who had no previous cataract surgery in their fellow eyes were included in the study. Verbal pain scale and visual analog scale were used to measure pain intensity. Demographic characteristics, concomitant systemic diseases, drug consumption, need of additional anesthesia during surgery, surgical complications, duration of surgery and surgeon comfort were also evaluated for each patient.

**Results:**

Seventy-two patients (78.3%) reported pain during surgery and 68 patients (73.9%) reported pain in the period after the surgery. When the intensity of pain during the surgery was evaluated, the percentage of patients reporting mild, moderate and intense pain was 35.9%, 25.0% and 17.4%, respectively. The average verbal pain score during the surgery was 1.4±1.0 (0-3). Reported pain level was not associated with age or gender (p>0.05). Diabetic patients and patients who consumed nonsteroidal anti-inflammatory drugs in the morning before operation reported less pain during and after the surgery (p<0.05). There were no complications except posterior capsule rupture in one patient. Duration of surgery was longer in patients who reported pain during surgery (p<0.05). There was no significant difference between pain reported during surgery and surgeon comfort (p>0.05).

**Conclusion:**

Patients frequently experience pain during phacoemulsification with topical anesthesia. Although pain perception does not affect surgical success, preoperative administration of analgesics in suitable patients or giving additional anesthesia to patients reporting severe pain during surgery may increase patient comfort.

## INTRODUCTION

Phacoemulsification under local anesthesia is the surgical method currently used to treat most cases of senile cataract.^1^ Retrobulbar or peribulbar anesthesia plus topical anesthesia (alone or with intracameral anesthesia) are commonly used for local anesthesia. Various potentially sight-threatening complications have been reported related to agents used in retrobulbar or peribulbar anesthesia or arising from the technique itself.^2^ Among these complications are chemosis, ecchymosis, retrobulbar hemorrhage, globe penetration or perforation, extraocular muscle damage, ptosis, amaurosis, penetration of the optic nerve sheath and optic atrophy.^3^ The use of topical anesthesia may help avoid possible complications of retrobulbar or peribulbar anesthesia, but the possibility of eye closing and movement due to pain sensation may present a significant handicap.

Topical anesthesia is growing in popularity with the clear corneal incision phacoemulsification procedure.^4^ Although the use of topical anesthesia during phacoemulsification surgery is a faster method and eliminates the risk of complications associated with local anesthesia, it has been reported that patients experience intraoperative and/or postoperative pain at a rate of 34 to 90%.^5,6,7^ In most studies the intraoperative and/or postoperative pain is reported as mild, but in some patients the pain is severe enough to require intervention and lasts for days.^5,6^

There are many reports in the literature about the effect of different anesthetic agents,^8^ additional sedation^9^ and preoperative analgesic medication^10^ on intraoperative and postoperative pain during cataract surgery under topical anesthesia. However, there are very few studies evaluating pain levels and the factors generally associated with pain. Awareness of the various factors that may affect pain could help identify patients with a greater chance of experiencing pain prior to the procedure, thus enabling necessary precautions to be taken and allowing a more comfortable and less complicated surgery. In this study, we aimed to evaluate patients’ intraoperative and postoperative pain using two commonly applied pain assessment methods, the verbal pain scale (VPS) and visual analog scale (VAS),^10,11^ and investigate the factors that may influence pain perception.

## MATERIALS AND METHODS

This prospective, single-centered, observational study included 92 patients who were evaluated in the Mevlana University Faculty of Medicine Ophthalmology Clinic and scheduled for routine clear corneal phacoemulsification surgery under topical anesthesia for senile cataract. It has been reported in the literature that pain occurs more often in a patient’s second cataract surgery; therefore, in this study we included patients undergoing cataract surgery for the first time.^12^ Patients with allergy or contraindication to the drugs likely to be used during the surgery, dementia, major psychiatric disorder or other neurological disease impacting memory and cognitive function; patients with only one eye or with visual acuity in the fellow eye too low to use the VAS; and patients who did not sign the informed consent form or were noncompliant were excluded from the study. Patients with any previous ocular surgery other than cataract surgery or any ocular disease such as glaucoma, uveitis or keratoconus were also excluded. Prior to the start of the study, approval was obtained from the local ethics committee, the study participants were informed about the study and informed consent forms were obtained. The study was conducted in accordance with the principles set forth in the Declaration of Helsinki.

Patients underwent a full ophthalmologic examination including visual acuity assessment, slit-lamp examination, applanation tonometry and fundus examination. A detailed medical history was obtained from each patient and concomitant systemic diseases and medications used regularly were recorded. Patients using warfarin were referred to the cardiology clinic before surgery to have their medication adjusted. Patients were instructed to take all regularly scheduled medications except aspirin on the morning of the operation and the VPS and VAS to be applied preoperatively were explained.

All patients underwent clear corneal phacoemulsification under topical anesthesia. Topical anesthesia consisted of 0.5% proparacaine hydrochloride (Alcaine 0.5%, Alcon Pharmaceuticals, Puurs, Belgium) drops applied to the ocular surface twice with a 2-3 minute interval. Intracameral anesthesia was not used. Prior to the surgery, the ocular surface was washed with 5% povidone iodine. All procedures were performed through clear corneal incisions with a Whitestar Signature (Abbott Medical Optics, Santa Ana, CA, USA) phacoemulsification system using the ‘stop and chop’ technique. The procedure was concluded by applying of 1 mg/0.1 ml cefuroxime to the anterior chamber and checking the incisions for leakage.

**Figure 1:**
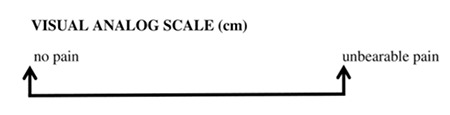
The visual analog scale

Patients’ pain levels were assessed intraoperatively and postoperatively using the VPS and VAS. On the VPS, pain is graded as 0 (no pain), 1 (mild pain), 2 (moderate pain), 3 (severe pain), or 4 (unbearable pain). Patients were asked to rate their level of pain with the VPS during the procedure, immediately following the procedure, and at 30 minutes, 1 hour, 2 hours, 4 hours and 24 hours after the procedure. For the VAS, patients were asked to use a pen to mark their level of pain on a 10 cm horizontal line on which one end was labeled ‘no pain’ (I feel no pain) and the other end was labeled ‘unbearable pain’ (The worst pain I have ever experienced) (Figure 1). The distance from the ‘no pain’ end of the line to the patient’s mark was measured in cm. Patients were asked to rate their level of pain with the VAS immediately following the procedure and at 30 minutes, 1 hour, 2 hours, 4 hours and 24 hours after the procedure. Patients were monitored in the hospital for 4 hours following the procedure. Patients’ demographic characteristics, concomitant systemic diseases, medications used (including nonsteroid anti-inflammatory [NSAI] drugs), additional anesthesia applied during surgery, surgical complications, duration of surgery (min) and surgeon comfort/discomfort were also evaluated. Cases in which the surgeon had to warn the patient or experienced difficulty due to the patient moving and/or tensing the eye were considered ‘surgeon discomfort’; the patient avoiding eye movement was considered ‘surgeon comfort’.

### Statistical Analysis

Data were statistically analyzed using SPSS v15.0 for Windows (SPSS, Inc.). The chi-square test was used for intergroup comparisons of nominal data and the Student’s t-test was used to compare numerical parameters. The level of significance was accepted as a=0.05.

## RESULTS

The study included 92 patients, 47 (51.1%) men and 45 (48.9%) women. The mean age of the patients was 66.9±10.6 (range, 43-85) years. There was no statistically significant difference between the mean ages of the male and female patients (men: 67.1±9.9 years, women: 66.8±11.4 years, p=0.89).

Seventy-two (78.3%) of the patients reported intraoperative pain and 68 (73.9%) reported postoperative pain. On intraoperative pain assessment using the VPS, 21.7% of the patients reported having no pain, while 35.9%, 25.0% and 17.4% of patients reported mild, moderate and severe pain, respectively. Immediately after the surgery, 26.1% of patients reported having no pain, whereas mild pain was reported by 32.6%, moderate pain by 22.8% and severe pain by 18.5% of the patients. None of the patients described their intra- or postoperative pain as unbearable. Seventy-two patients (78.3%) reported feeling no pain after the second postoperative hour. The patients’ intraoperative and postoperative pain levels evaluated by VPS and VAS are shown in Table 1.

No statistically significant association was found between reported intraoperative and postoperative pain levels and age or gender (p>0.05) (Table 2). Concomitant systemic diseases were present in 62% of the patients in the study. Diabetic patients (n=28) reported significantly less pain both intra- and postoperatively (p=0.03 and p=0.01, respectively). No significant associations emerged between other systemic diseases and pain scores (p>0.05). Patients who took an NSAI drug on the morning of the surgery (42 patients, 45.6%) reported significantly less intraoperative pain (p<0.001). However, after the second postoperative hour, there was no difference in pain level between patients who took an NSAI drug and those who did not (Table 3).

Additional anesthesia (intravenous fentanyl) was administered to 3 patients who tensed or moved their eyes because of intraoperative pain. Posterior capsule rupture occured in one of the patients included in the study. This patient reported moderate to severe pain. None of the patients developed serious postoperative complications. 

The mean surgery duration was 22.2±4.6 minutes. Surgery duration was significantly longer in patients who reported having intraoperative and postoperative pain (p=0.003 and 0.01, respectively). Among patients with no intraoperative pain, the surgeon’s comfort was rated as good in 80%, while the rate of good surgeon comfort was 62.5% among patients with intraoperative pain. However, the difference was not statistically significant (p=0.14).

## DISCUSSION

Many studies have demonstrated the efficacy and safety of cataract surgery through clear corneal incision under topical anesthesia, and it is one of the most common surgical procedures performed today.^13,14,15^ Topical anesthesia is preferred over local anesthesia by patients because it does not require an injection like in the retrobulbar or peribulbar application of local anesthesia, and also by physicians because it avoids injection-related complications.

In the current study evaluating pain levels experienced by patients undergoing phacoemulsification under topical anesthesia, 72 of 92 patients (78.3%) reported feeling pain intraoperatively. In a recent study investigating the analgesic efficacy of topical anesthesia for phacoemulsification surgery, pain was reported by 89.5% of the patients.^6^ In another study including 124 eyes of 96 patients, pain was reported at a rate of 71.8% among patients undergoing cataract surgery under topical anesthesia. However, in this study, deep topical anesthesia was achieved using topical anesthetic drops as well as sponges soaked in an anesthetic substance applied to the inferior and superior fornices.^13^

In the current study, a total of 68 patients (73.9%) reported postoperative pain, mostly within the first hour after surgery. Porela-Tiihonen et al.^5^ evaluated patients’ postoperative pain levels after phacoemulsification with topical anesthesia and reported that 34% of their patients felt pain postoperatively, with 10% of patients describing their pain as severe the day after surgery. The authors stated that patients were asked to rate their pain within the first 4 hours after the procedure. The lack of a specific time when patients’ pain levels were assessed may prevent the proper evaluation of these data. In our study, 60.9% of patients reported having pain at 30 minutes after the surgery, whereas 21.7% reported pain at postoperative 2 hours.

Many studies have utilized the VPS and/or VAS to evaluate pain levels during phacoemulsification surgery under topical anesthesia. Apil et al.^^6^^ used the VPS with patients who received topical anesthesia and found a mean pain level of 1.01±0.41. In the current study, the intraoperative pain level was evaluated as 1.4±1.0 using the VPS. In their studies comparing topical anesthesia to sub-Tenon’s anesthesia, Srinivasan et al.^16^ reported VPS pain levels of 3.44±2.3 and 2.25±2.2 and Zafirakis et al.^17^ of 1.13±1.57 and 0.80±0.93 in the topical anesthesia group during or immediately after the surgery and at postoperative 30 minutes, respectively. The mean pain level as assessed by VPS in the current study was 2.5±2.2 immediately after surgery and 1.6±1.8 at postoperative 30 minutes.

We detected no association between intra- or postoperative pain levels and patient age or gender. Similarly, Apil et al.^6^ reported that intraoperative pain was not associated with age or gender in their study. However, in a study including 506 patients, Tan et al.^18^ found that female patients experienced more pain during cataract surgery, while Gombos et al.^19^ reported that young patients were more sensitive to pain during cataract surgery.

Most patients scheduled for senile cataract have one or more concomitant systemic diseases. To the best of our knowledge, there are no studies in the literature investigating the relationship between concomitant diseases and pain sensation during cataract surgery. We found that diabetic patients reported feeling less pain during and after cataract surgery. This may be attributable to diabetic neuropathy. Mocan et al.^20^ observed by confocal scanning laser microscopy that the corneal nerve plexus was less dense and showed more morphologic abnormalities in diabetic patients.

We found that surgery duration was significantly longer in patients who reported having intraoperative and postoperative pain. Rothschild et al.^7^ also reported that surgery duration was significantly longer in the patient group with high pain scores. No relation emerged in our study between intraoperative pain and surgeon comfort. It has been reported in the literature that the use of sedation in addition to topical anesthesia resulted in less pain and better surgeon comfort.^9^

Although topical anesthesia does not provide analgesia as effectively as retrobulbar or peribulbar anesthesia, there is evidence that these methods do not differ in terms of surgical outcome and reliability.^4^ However, as topical anesthesia does not affect the intraocular tissues, patients may feel pain in certain situations, such as when the surgical instrument touches the iris, and blood pressure, heart rate and serum adrenaline levels may rise as a result.^19^ Various techniques such as intracameral anesthesia,^15^ supplemental sedation^9^ and taking an analgesic preoperatively^10^ have been shown to be effective in addition to topical anesthesia for improving surgical safety and comfort. We also found that patients who used an NSAI drug on the morning of the surgery had significantly lower pain levels.

In the current study, most of the patients undergoing phacoemulsification surgery under topical anesthesia experienced pain. However, most patients’ pain was mild to moderate, and pain perception was not associated with surgical outcome or surgeon comfort.

## CONCLUSION

Most patients experience pain during phacoemulsification surgery under topical anesthesia. Although pain perception does not affect surgical success, giving selected patients an analgesic prior to surgery or administering additional anesthesia to patients with severe pain can improve patient comfort.

### Ethics

Ethics Committee Approval: Mevlana University Ethic Committee Approval Number 2013/004, Informed Consent: Obtained.

Peer-review: Externally peer-reviewed.

## Figures and Tables

**Table 1:**
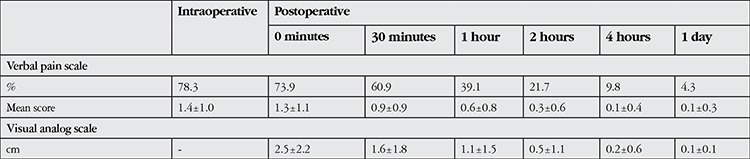
Patients’ mean pain levels assessed using the verbal pain scale and visual analog scale

**Table 2:**
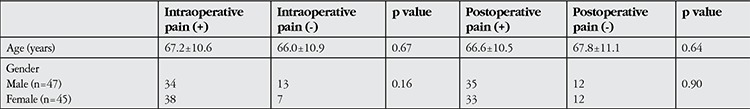
Comparison of the demographic characteristics of patients with and without intraoperative and postoperative pain

**Table 3:**
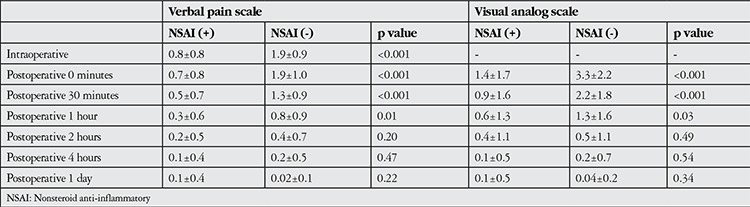
Comparison of mean pain levels assessed by verbal pain scale and visual analog scale (cm) according to nonsteroid anti-inflammatory use
